# Genome‐wide association analysis reveals genes controlling an antagonistic effect of biotic and osmotic stress on *Arabidopsis thaliana* growth

**DOI:** 10.1111/mpp.13436

**Published:** 2024-03-09

**Authors:** Pingping Huang, Mohamed El‐Soda, Katarzyna W. Wolinska, Kaige Zhao, Nelson H. Davila Olivas, Joop J. A. van Loon, Marcel Dicke, Mark G. M. Aarts

**Affiliations:** ^1^ Laboratory of Genetics Wageningen University & Research Wageningen Netherlands; ^2^ Department of Genetics, Faculty of Agriculture Cairo University Giza Egypt; ^3^ Laboratory of Entomology Wageningen University & Research Wageningen Netherlands; ^4^ Present address: Shenzhen SinoPlant Biotech Ltd Dapeng Marine Organism Industrial Park, Gongye Ave, Dapeng District 518000 Shenzhen China.; ^5^ Present address: College of Horticulture and Forestry Huazhong Agriculture University Nanhu Road, Hongshan District 430070 Wuhan China.; ^6^ Present address: BASF Vegetables Seeds Napoleonsweg 152 Nunhem 6083 AB Netherlands.

**Keywords:** (a)biotic stress response, *Arabidopsis thaliana*, combinatorial stress, genome‐wide association mapping, genotype‐by‐environment interaction, QTL × E

## Abstract

While the response of *Arabidopsis thaliana* to drought, herbivory or fungal infection has been well‐examined, the consequences of exposure to a series of such (a)biotic stresses are not well studied. This work reports on the genetic mechanisms underlying the *Arabidopsis* response to single osmotic stress, and to combinatorial stress, either fungal infection using *Botrytis cinerea* or herbivory using *Pieris rapae* caterpillars followed by an osmotic stress treatment. Several small‐effect genetic loci associated with rosette dry weight (DW), rosette water content (WC), and the projected rosette leaf area in response to combinatorial stress were mapped using univariate and multi‐environment genome‐wide association approaches. A single‐nucleotide polymorphism (SNP) associated with *DROUGHT‐INDUCED 19* (*DI19*) was identified by both approaches, supporting its potential involvement in the response to combinatorial stress. Several SNPs were found to be in linkage disequilibrium with known stress‐responsive genes such as *PEROXIDASE 34* (*PRX34*), *BASIC LEUCINE ZIPPER 25* (*bZIP25*), *RESISTANCE METHYLATED GENE 1* (*RMG1*) and *WHITE RUST RESISTANCE 4* (*WRR4*). An antagonistic effect between biotic and osmotic stress was found for *prx34* and *arf4* mutants, which suggests *PRX34* and *ARF4* play an important role in the response to the combinatorial stress.

## Graphical abstract

The combination of *Arabidopsis* exposure to *Pieris rapae* herbivory and polyethylene glycol (PEG) osmotic stress particularly effects the growth rates of particular mutants based on the slope of the increase in projected leaf area.

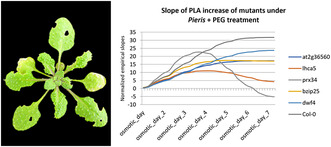

## 1 INTRODUCTION

Plant responses to biotic and abiotic stresses are mainly regulated by phytohormones. For example, plant tolerance to abiotic stresses such as drought and salt are mainly regulated through the phytohormone abscisic acid (ABA) signalling pathway (Berens et al., 2019; Shinozaki & Yamaguchi‐Shinozaki, 2007). In case of biotic stresses, jasmonic acid (JA) and ethylene (ET) are the main factors regulating plant responses to necrotrophic fungi and chewing insects, while salicylic acid (SA) activates plant defence responses upon infection with biotrophic fungi (Dodds & Rathjen, 2010; Pieterse et al., 2009).

Generally, plant response to an individual stress is different from its response to combinatorial or consecutive exposures of biotic and abiotic stresses, which trigger specific multiple stress‐responsive gene expression (Atkinson & Urwin, 2012; Berens et al., 2019; Pandey et al., 2017; Rasmussen et al., 2013; Sewelam et al., 2014). For instance, Rasmussen et al. (2013) showed that 61% of the transcription changes in response to a combinatorial stress could not be predicted from the single stress responses. Prasch and Sonnewald (2013) showed that in *Arabidopsis thaliana* (*Arabidopsis*), the expression of the resistance gene *RPS6* was only induced in case of simultaneous heat, drought and virus treatments, but not under any of the double or individual stresses. Transcriptome analysis on simultaneous effects of drought, heat and virus infections in *Arabidopsis* resulted in identifying 23 genes that were specifically regulated by the triple stress treatments (Prasch & Sonnewald, 2013).

In nature, plants are simultaneously or sequentially exposed to various biotic and abiotic stress‐inducing factors that limit plant performance and cause a substantial annual reduction in agricultural production (Berens et al., 2019; Hirt, 2004; Pandey et al., 2017). The interaction between biotic and abiotic stress responses is often mediated by different plant hormones such as ABA, JA, ET, SA and the outcome can be either antagonistic, synergistic or neutral (Fujita et al., 2009; Pareek et al., 2010; Pieterse et al., 2012). Antagonistic interactions have been observed between JA and SA, ABA and SA, ABA and JA, and ABA and ET, while synergistic interactions have been observed between ABA and auxin (AUX) (Yoshioka & Shinozaki, 2009).

Significant effects of abiotic stresses on plant responses to biotic stresses, and vice versa, were observed previously (Appel et al., 2014; Atkinson & Urwin, 2012; Berens et al., 2019; Rejeb et al., 2014). For instance, drought‐increased ABA concentrations have a positive impact on callose deposition, which may boost plant resistance to fungal and bacterial pathogens (Achuo et al., 2006; Mauch‐Mani & Mauch, 2005). Turnip mosaic virus causes lesions, mosaics and mottling that reduce photosynthetic capacity, thereby rendering the plant more susceptible to subsequent drought stress (Prasch & Sonnewald, 2015).

Most studies on the response of plants to multiple stresses have only focused on a limited number of genotypes (Narsai et al., 2013; Ramegowda & Senthil‐Kumar, 2015; Sewelam et al., 2014; Shaik & Ramakrishna, 2013), which is fine to understand the general physiology of the response, but insufficient to understand the genetic architecture of such responses. For *Arabidopsis* it is attractive to investigate the natural genetic variation of a trait using genome‐wide association (GWA) mapping, which gives high genetic resolution for traits with high heritability (Bac‐Molenaar et al., 2015; Baxter et al., 2010; Chao et al., 2012; El‐Soda et al., 2019; El‐Soda & Sarhan, 2021; Feng et al., 2022; Li et al., 2010; Ogura & Busch, 2015; Zhu et al., 2021).

This research was conducted to study the broad genetic basis underlying the consecutive biotic and abiotic stress responses and complements previous work on other combinations of biotic and abiotic stress in *Arabidopsis* (Coolen et al., 2016, 2022; Davila Olivas et al., 2016, 2017; Thoen et al., 2017). *Arabidopsis* was used as a model organism to study the responses to different consecutive combinations of biotic factors, namely, infection with *Botrytis cinerea* (*Botrytis*) or herbivory by *Pieris rapae* (*Pieris*) and osmotic stress, as an abiotic factor. Plants that were exposed to *Pieris* larvae or to the necrotrophic fungus *Botrytis* are expected to trigger JA‐ET‐mediated signalling either through the MYELOCYTOMATOSIS ONCOGENE (MYC)‐regulated pathway (Lorenzo et al., 2004), which is co‐regulated by drought‐induced ABA (Abe et al., 2003) or through the ETHYLENE RESPONSE FACTOR (ERF)‐regulated pathway (co‐regulated by ET), which is antagonistically regulated by ABA (Pré et al., 2008; Vos et al., 2013). Therefore, an enhanced tolerance to the subsequent stress combination of *Pieris* and drought and a reduced tolerance to the sequential stress combination of *Botrytis* and drought are expected.

Plants may try to escape from drought by closing stomata and reducing photosynthesis, thereby retaining water inside the plant or by enhancing the root system, to acquire more water (Verslues et al., 2006). Physiological responses to drought include reduced rosette dry weight (DW), reduced rosette water content (WC) and reduced projected rosette leaf area (PLA) (Clauw et al., 2016; Hummel et al., 2010). Several studies have showed that exposure to an osmoticum, such as polyethylene glycol (PEG), affects plant growth in a similar way to withholding water, and can be used to impose drought stress‐like symptoms (Alqurashi et al., 2018; Pandey et al., 2017). In the present study, *Arabidopsis* plants were either exposed to *Pieris* larvae or to the necrotrophic fungus *Botrytis*, followed by PEG treatment. This study therefore focuses on the effect of a biotic stress pretreatment on sequential osmotic stress in plants. The three described phenotypic traits were used to record the *Arabidopsis* response to combined stresses. The broad genetic base of the response was examined by genome‐wide association studies (GWAS) using 341 *Arabidopsis* natural accessions. In this way, we could identify single‐nucleotide polymorphisms (SNPs) associated with the studied phenotypes, representing many quantitative trait loci (QTLs) illustrating the *Arabidopsis* response to these combined stresses and the genotype‐by‐environment interactions affecting the phenotypes. Furthermore, we could suggest several candidate genes for which allelic variation associates with the observed phenotypic variation and provide further support for some of these candidate genes by characterizing the phenotypes of T‐DNA insertion mutants under the stress‐inducing conditions.

## 2 RESULTS

### 2.1 Drought stress response can be mimicked by exposure to polyethylene glycol

**FIGURE 1:**
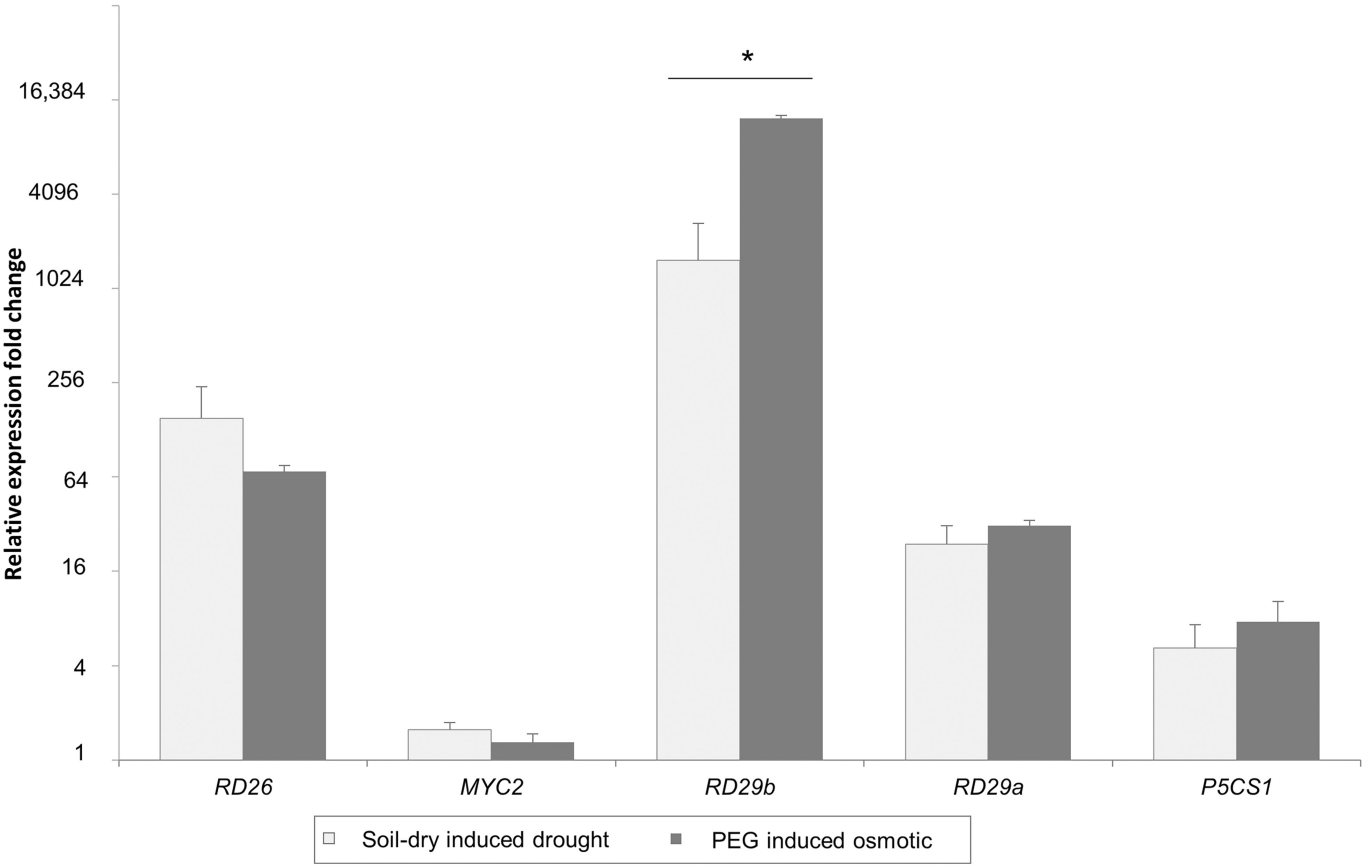
Relative expression fold changes of five drought‐responsive genes *RD26*, *MYC2*, *RD29b*, *RD29a* and *P5CS1* in rosettes of *Arabidopsis* Col‐0 plants, either grown on a peat‐based mixture, subjected to drought by withholding water (white), or grown on rock wool, watered with a nutrient solution containing polyethylene glycol 8000 (PEG), to induce an osmotic stress response (grey). Relative gene expression fold change was determined by comparing expression in treated plants with expression in plants growing under well‐watered conditions (either growing in a well‐watered peat mixture or on rock wool watered with a nutrient solution without PEG), acting as control. Student's *t* test was used to compare expression of each gene in the different conditions. *p < 0.05. Standard errors were calculated based on at least four plants per treatment.

To induce a stress response mimicking the response to drought, a PEG 8000 treatment was applied. *Arabidopsis* plants exposed to 6 days of the PEG treatment appeared very similar to plants grown on a peat–sand mixture left without water for 6 days. However, to show that the transcriptional response of *Arabidopsis* to PEG and to drought by withholding water was also comparable, the expression of five genes involved in the ABA‐dependent or the ABA‐independent drought response pathway response (Shinozaki & Yamaguchi‐Shinozaki, 2007) was determined in plants subjected to either treatment (Figure 1). Except for *RD29b*, the change in the expression of these genes compared with the control conditions was similar after the two treatments. Even though the expression of *RD29b* was slightly, but significantly, more induced in the PEG treatment than in the drought treatment, we considered the PEG treatment to acceptably mimic the drought treatment.

### 2.2 Phenotyping the HapMap population under the single and the combinatorial stress

**FIGURE 2:**
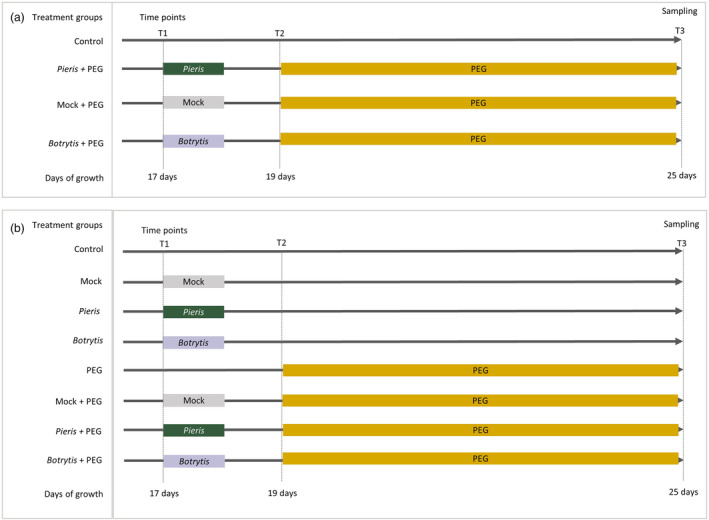
Experimental design for the treatment of *Arabidopsis* plants subjected to control conditions (dark grey arrow) or to a *Pieris rapae* herbivory treatment (green), to *Botrytis cinerea* infection (purple), a mock treatment with the solution in which no *Botrytis* spores were suspended (light grey), to a polyethylene glycol 8000 (PEG) treatment (yellow), or to the described treatment combinations. Vertical arrows indicate sampling time points (T1, T2, T3, Sampling). *Pieris*, *Botrytis* or mock treatments were applied for 1 day, the PEG treatment started 1 day after ending the first treatments. Design in (a) was used for genome‐wide association mapping, design in (b) for T‐DNA mutants and extreme accessions of the HapMap population.

The *Pieris* treatment and the *Botrytis* treatment were introduced to the *Arabidopsis* HapMap population to induce either herbivory priming or fungal infection before exposure to osmotic stress using PEG (Figure 2). Rosette fresh weight (FW) and PLA were determined in the *Pieris* treatment, while in the *Botrytis* treatment, FW, PLA, rosette dry weight (DW) and water content (WC) were measured. The FWs of plants exposed to different treatments were all significantly correlated (Table S3). Broad‐sense heritability (*H* ^2^) was calculated for the measured traits for control and treated plants, which ranged from 0.37 to 0.63 (Table S4). Two‐way analysis of variance (ANOVA) was performed to test the interaction between treatments and accessions for PLA, FW, DW and WC. Significant treatment and accession effects were found, but no significant interactions between accessions and treatments (Table S5).

### 2.3 Univariate GWA‐mapping of residuals identified SNPs associated with phenotypic plasticity

**FIGURE 3:**
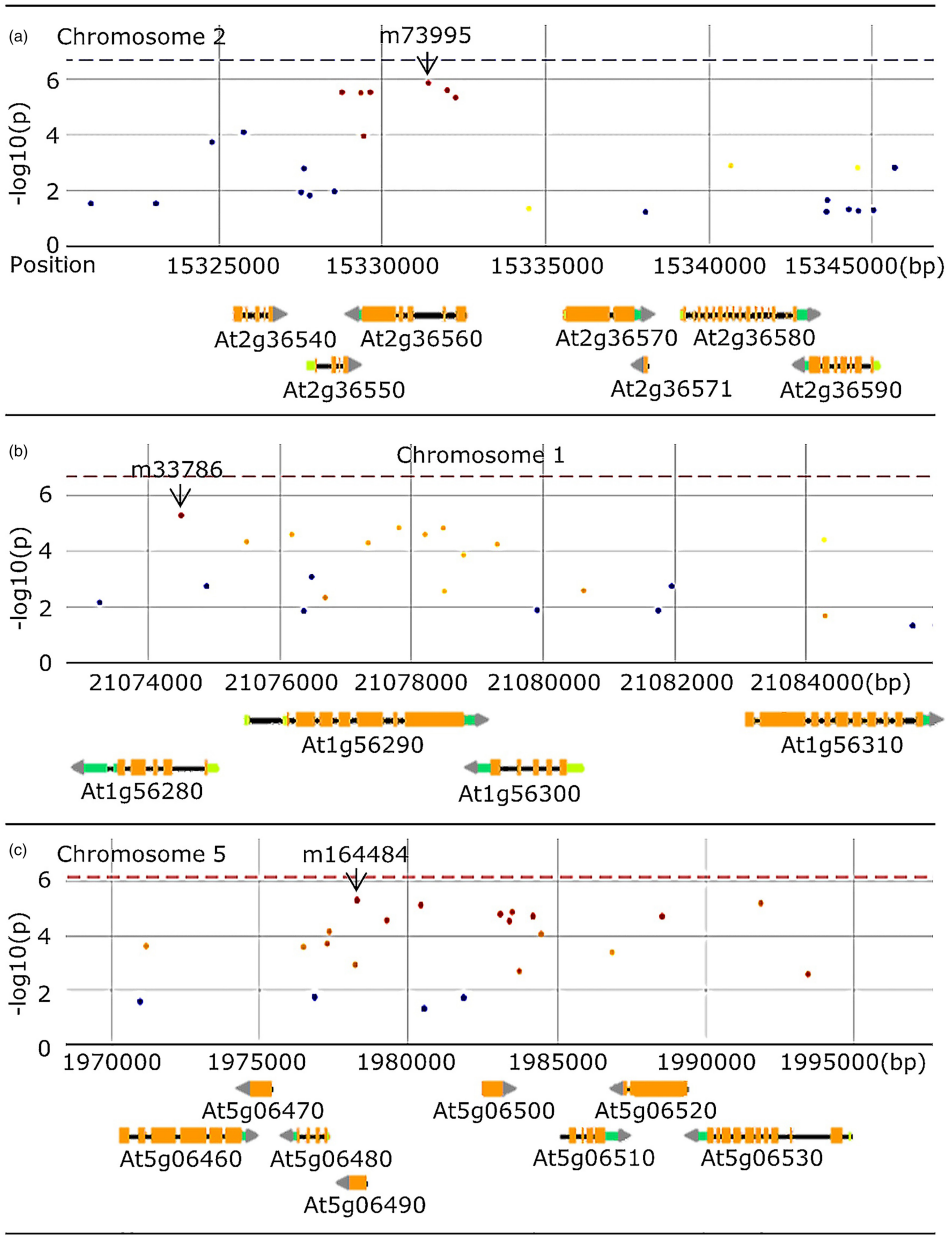
Genomic regions of (a) chromosome 2, between 15,324,282 and 15,346,000 bp, containing genes At2g36540–At2g365090, with 11 single‐nucleotide polymorphisms (SNPs) in the region being associated with projected leaf area (PLA) of *Arabidopsis* rosettes in response to a *Pieris* + polyethylene glycol 8000 (PEG) combined treatment (Table S7); (b) chromosome 1, between 21,072,000 and 21,088,000 bp, containing genes At1g56280–At1g56330, with nine SNPs in the region being associated with PLA of *Arabidopsis* rosettes in response to a *Botrytis* + PEG combined treatment (Table S7); (c) chromosome 5, between 1,970,000 and 2,005,000 bp, containing genes At5g06490–At5g06530, with nine SNPs in the region being associated with water content of *Arabidopsis* rosettes in response to a *Botrytis* + PEG combined treatment (Table S7). The SNPs associated with the highest −log_10_(*p*) value for each locus are indicated: m73995 (a), m33786 (b) and m164484 (c). SNPs indicated in red are in high linkage disequilibrium (LD) (LD >0.8) with the SNP that exhibited the highest –log(*p*) score among the SNPs. SNPs indicated in yellow are in lower LD (0.3 < LD <0.8) with the m33786, m73995 or m164484 SNP. The horizontal dashed line indicates the conservative Bonferroni −log_10_(*p*) threshold for multiple testing assuming full independence of SNPs. Gene models are indicated with black arrows, with yellow boxes indicating exons and light green and blue‐green boxes indicating the 5′ and 3′ untranslated regions. Genome‐wide association mapping results were obtained using an accelerated mixed model (AMM) method from online tool GWAPP (https://gwas.gmi.oeaw.ac.at).

**FIGURE 4:**
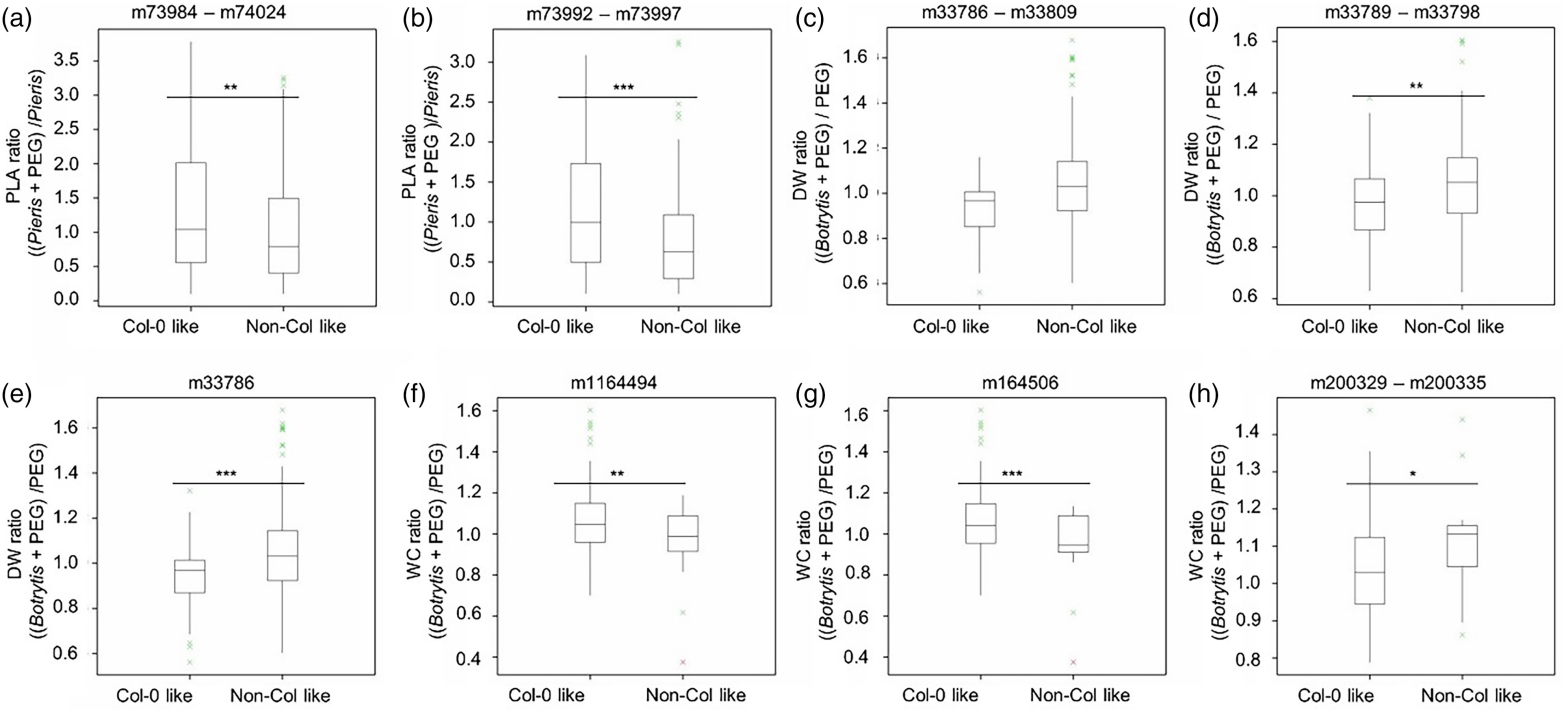
Box plots showing the effect of (combinations of) Col‐0 and non‐Col‐0 alleles on indicated traits. The combinations are shown of (a) 11 associated single‐nucleotide polymorphisms (SNPs) (m73984–m74024) and (b) 6 associated SNPs (m73992–m73997), residing in At2g36560, for the rosette project leaf area (PLA) ratios comparing the *Pieris* + polyethylene glycol 8000 (PEG) treatment versus the single *Pieris* treatment. Furthermore of (c) nine SNPs (m33786–m33809), (d) six SNPs (m33789–m33798 (residing in At1g56290) and (e) one SNP (m33786) (residing in *DI19*) on rosette dry weight (DW) ratios comparing the *Botrytis* + PEG treatment versus the single PEG treatment. And finally of single SNPs (f) m1164494, (g) m164506 and (h) m200329–m200335 on rosette water content (WC) ratios comparing the *Botrytis* + PEG treatment versus the single PEG treatment. Student's *t* test was used to determine the significance of the phenotypic difference between accessions with either one of the two types of alleles, with *p < 0.05, **p < 0.01, *** *p* < 0.001.

To observe the effect of *Pieris* priming on plant growth in response to subsequent PEG, residuals were calculated from a regression of PLA of the *Pieris* and PEG stress treatment on PLA of the *Pieris* treatment alone. When these PLA residual values were used for GWA analysis, 60 associated SNPs were mapped to 47 genes (Figure S1a, Table S6). Eleven SNPs were mapped to a genomic region that spans only 19 kb on chromosome (Chr) 2, in which six genes, At2g36540–At2g36590, are located (Figure 3a). Two haplotypes were distinguished for the associated SNPs in this region that were in linkage disequilibrium (LD), which either corresponded to a Col‐0 or a non‐Col‐0 allele. The combined phenotype boxplot of the haplotypes, combining 9 of these 11 SNPs, showed that accessions containing the non‐Col‐0 allele had comparably lower rosette PLA under the *Pieris* and PEG treatment versus the single *Pieris* treatment than accessions containing the Col‐0 allele (Figures 4a and S2). Six significant SNPs located in At2g36560 were associated with PLA residuals. Significant reduction in PLA was observed comparing accessions carrying the non‐Col allele to the accessions carrying the Col‐0 allele (Figure 4b).

When the residuals resulting from the regression of the DW upon *Botrytis* and PEG treatment on the DW upon PEG treatment alone were used for GWA mapping, 26 associated SNPs residing in 17 genes were identified (Figure S1b, Table S6). Nine of these SNPs mapped to a locus spanning 9.7 kb of Chr 1, corresponding to four genes, At1g56280–At1g56310 (Figure 3b). The combined boxplot of the haplotypes combining these nine SNPs (m33786–m33809) showed that accessions containing the non‐Col‐0 allele had comparably higher rosette DW under the *Botrytis* + PEG treatment versus the single PEG treatment than accessions containing the Col‐0 allele (Figure 4c,d). The SNP marker m33786 mapped in the *DROUGHT‐INDUCED 19* (*DI19*) gene (At1g56280). The allelic effect of the SNP m33786 in *DI19* gene is shown in Figure 4e and significantly distinguishes accessions carrying the non‐Col‐0 allele from those carrying the Col‐0 allele, with the former allele contributing to a relatively higher DW in response to the combined treatment.

Mapping the residuals for WC, comparing the combined *Botrytis* and PEG treatment with the single PEG treatment, identified 24 associated SNPs, corresponding to 18 genes (Figure S1c, Table S6). A subset of these reside in a region of 14.5 kb on Chr 5 comprising At5g06480–At5g06530 (Figure 3c). One of these genes is *ATP‐BINDING CASSETTE TRANSPORTER G22* gene (At5g06530), mutation of which is known to cause increased water transpiration and drought susceptibility (Kuromori et al., 2011). Accessions carrying the non‐Col‐0 haplotype for SNP markers m164484 and m164506, which characterize this region on Chr 5, had a lower relative WC upon *Botrytis* and PEG stress versus PEG stress alone, when compared to accessions carrying the Col‐0 allele (Figure 4f,g).

### 2.4 Multi‐environment GWA‐mapping identified SNPs interacting with the stress treatments

Next to the univariate GWA‐mapping approach, we also employed multi‐environment (ME) GWA‐mapping using a multi‐trait mixed model (MTMM) approach (El‐Soda et al., 2015; Korte et al., 2012). This method was used to map significant SNPs associated with PLA measured at the two time points (T), with T1 representing the *Pieris* pretreatment and T2 representing the *Pieris* and PEG treatment (Figure 2). This analysis revealed 39 significant SNPs (Figure S1d, Table S7), of which seven SNPs were mapped to a region of Chr 2 containing two genes, At2g36550 and At2g36560, that were also found to be associated with PLA using the univariate GWA analysis (Figure S1a). Similar to the univariate results, the combined boxplot of the haplotypes combining the seven SNPs showed that accessions carrying the non‐Col allele of At2g36560 exhibited reduced PLA under the *Pieris* and PEG treatment versus the single *Pieris* treatment when compared to accessions containing the Col‐0 allele (Figure 4b).

Mapping SNPs associated with the DW measured under the combined *Botrytis* and PEG treatment and the single PEG treatment revealed 10 associated SNPs that correspond to nine genes (Figure S1e, Table S7). One of these SNPs, m33786, was mapped to the intron of the *DI19* gene, also identified upon univariate GWA analysis (Figure 4c,e). Similar to the univariate results, the combined boxplot of the haplotype of this SNP showed that the non‐Col allele contributes to relatively higher DW, indicating increased tolerance to the consecutive combination of *Botrytis* and PEG stress in comparison with the Col‐0 allele (Figure 4e).

When mapping the significant SNPs associated with WC for the same treatments, 43 SNPs were identified, corresponding with 30 genes (Figure S1f, Table S7). Four of these SNPs were mapped to a region on Chr 3 containing seven genes, At3g22670–At3g22730, including the genes in LD with these SNPs. In addition, six associated SNPs were mapped to a region on Chr 5 containing seven genes, At5g48120–At5g48180, with SNPs m200317 and m200318 residing in the *MET18* gene (At5g48120), and SNP m200389 residing in the *NITRILE SPECIFIER PROTEIN 5* (*ATNSP5*) gene (At5g48180). Finally, associated SNP m60883 was identified in At2g13690 by univariate and ME GWA‐mapping approaches. The boxplot of the haplotypes combining the SNPs mapped in the five previous examples showed no significant difference in the WC ratio for the *Botrytis* and PEG treatment versus the single *Botrytis* treatment, when comparing accessions with the Col‐0 or non‐Col‐0 allele (Figure S3a–e). There is one exception: accessions with haplotypes combing the non‐Col‐0 alleles for the three SNPs residing in genes At5g48130 and At5g48140 (m200329, m200331 and m200335) exhibited a higher WC ratio for the *Botrytis* and PEG treatment versus the single *Botrytis* treatment, when compared to accessions containing the Col‐0 allele (Figure 4h).

Although only a fraction of all genetic polymorphisms between accessions are represented by the SNP markers used for GWA analysis, some of the associated SNPs might actually be causal for the observed allelic variation, as they correspond with non‐synonymous changes in the predicted open reading frames of the genes they reside in. Some of these altered amino acid sequences may affect the protein function. Examples of these are the SNP marker m73991 in At2g36550, a gene encoding a haloacid dehalogenase‐like hydrolase (HAD) superfamily protein, which corresponds with an Asp to Lys amino acid substitution (N138K) in the non‐Col‐0 versus the Col‐0 allele; and SNPs m73993 and m73994, which cause non‐synonymous changes to the coding region of At2g36560, encoding a protein of unknown function with very low expression, substituting Ala to Glu (A488E) and Glu to Gly (E420G).

### 2.5 *PRX34* and *ARF4* play a role in the response of PLA to consecutive biotic and PEG stresses

Several of the associated SNPs that were identified in either approach or that were in LD with such associated SNPs, correspond with genes that have been reported to play a role in the response to biotic or abiotic stresses. However, none of these have been reported to actually function in the response to a *Pieris*, *Botrytis* or PEG treatment. To establish if any of the identified *Arabidopsis* genes may be involved in the response to any of these stresses, we identified plants carrying a single homozygous T‐DNA insertion mutant allele of the *At2g36560*, *LCHA5*, *PEX34, bZIP25, DI19, DWF4, RMG1, WRR4* and *ARF4* genes (Table 1), and we determined their PLA phenotypes.

**TABLE 1:** Selection of eight genes associated with quantitative trait loci for the response to stress treatments, which were examined for their role in the stress response.

Treatment	Gene TAIR IDs	Gene name	Gene description	Associated SNP	LD score	−log_10_(*p*) of the associated marker
*Pieris* & PEG	At2g36560	Unnamed gene	A paternally expressed imprinted gene	m73994	1	6.28
*Pieris* & PEG	At1g45474	PHOTOSYSTEM I LIGHT‐HARVESTING COMPLEX GENE 5 (*LHCA5*)	Encodes a component of the light‐harvesting complex of photosystem I	m27735	1	4.12
*Pieris* & PEG	At3g49120	PEROXIDASE 34 (*PRX34*)	Class III peroxidase Perx34. Expressed in roots, leaves and stems. Located in the cell wall. Involved in cell elongation. Expression activated by light. May play a role in generating H_2_O_2_ during defence response	m114713	0.88	4.44
*Pieris* & PEG	At3g54620	ARABIDOPSIS THALIANA BASIC LEUCINE ZIPPER 25 (*bZIP25*)	bZIP transcription factor‐like protein	m118294	0.86	4.55
*Botrytis* & PEG	At1g56280	ARABIDOPSIS THALIANA DROUGHT‐INDUCED 19 (*ATDI19*)	Encodes a gene whose transcript level in root and leaves increases to progressive drought stress. The increase in transcript level is independent from abscisic acid level. It appears to be a member of plant‐specific gene family. It is phosphorylated by AtCPK11 in a Ca(^2+^)‐dependent manner at Thr105 and Ser107 within the AtDi19 bipartite nuclear localization signal.	m33786	1	5.35
*Botrytis* & PEG	At1g56510	WHITE RUST RESISTANCE 4 (*WRR4*)	TIR‐NB‐LRR protein that confers resistance to four races of *Albugo candida*	m33786	0.31	5.35
*Botrytis* & PEG	At4g11170	Resistance methylated gene 1 (*RMG1*)	Disease resistance protein (TIR‐NBS‐LRR class)	m137156	0.64	4.05
*Botrytis* & PEG	At5g60450	AUXIN RESPONSE FACTOR 4 (*ARF4*)	Encodes a member of the ARF family of transcription factors that mediate auxin response	m209212	0.51	4.32

*Note*: Gene descriptions and ID taken from The Arabidopsis Information Resource (TAIR, http://www.arabidopsis.org).

Abbreviations: LD, linkage disequilibrium; SNP, single‐nucleotide polymorphism.

**FIGURE 5:**
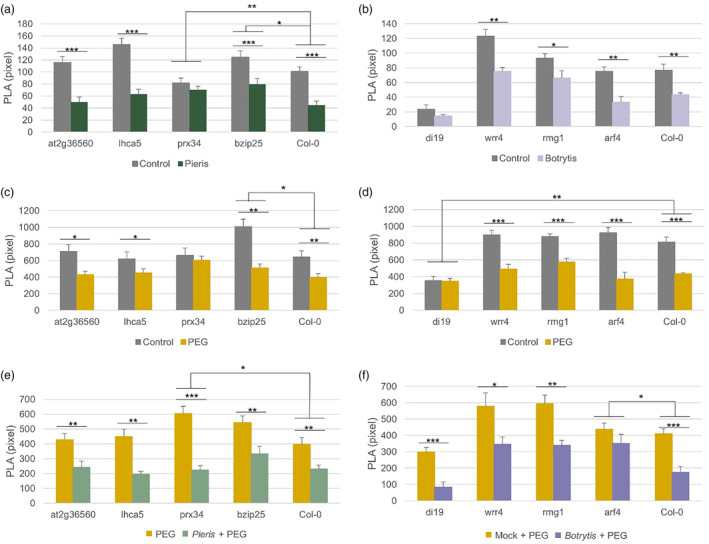
Ratios of projected rosette leaf areas (PLA) of T‐DNA insertion mutants of the HAD family gene At2g36560, *LHCA5, PRX34* and *bZIP25* (a, c, e), and *DI19*, *WRR4, RMG1* and *ARF4* (b, d, f), compared with Col‐0 wild type in response to (a) *Pieris* treatment versus control; (b) *Botrytis* treatment versus Mock; (c, d) polyethylene glycol 8000 (PEG) treatment versus control; (e) *Pieris* + PEG treatment versus PEG only treatment; (f) *Botrytis* + PEG treatment versus Mock + PEG treatment. Each sample comprised six plants per treatment per genotype. Two‐way analysis of variance was used for analysis of differences between mutants and the wild type. *p < 0.05, **p < 0.01, ***p < 0.001. *p*‐values of the main and interaction effects are provided in Table S8.

Mutant plants were subjected to the same treatments as were implemented for the HapMap accessions, as well as a few others not involving the PEG treatment (Figure 2b). One mutant, *di19*, exhibited a small rosette phenotype in all conditions when compared to the wild type (Figure S4). Two‐way ANOVA was performed to test the significance of the PLA differences (treatment vs. control) of T‐DNA insertion mutants compared with the wild type. The *prx34* and *bzip25* mutants were more tolerant to *Pieris* than the wild type (Figure 5a). When determining the response to PEG, all mutants showed significant reduced rosette area, especially the *bzip25* mutant, but not the *di19* mutant, compared with the wild type (Figure 5c,d). Regarding the combinatorial treatments, the *prx34* mutant was more sensitive to the *Pieris* + PEG treatment (Figure 5e), while the *arf4* mutant was more tolerant to the *Botrytis* + PEG treatment (Figure 5f).

### 2.6 Variation in candidate gene expression in extreme accessions grown under different stress conditions

**FIGURE 6:**
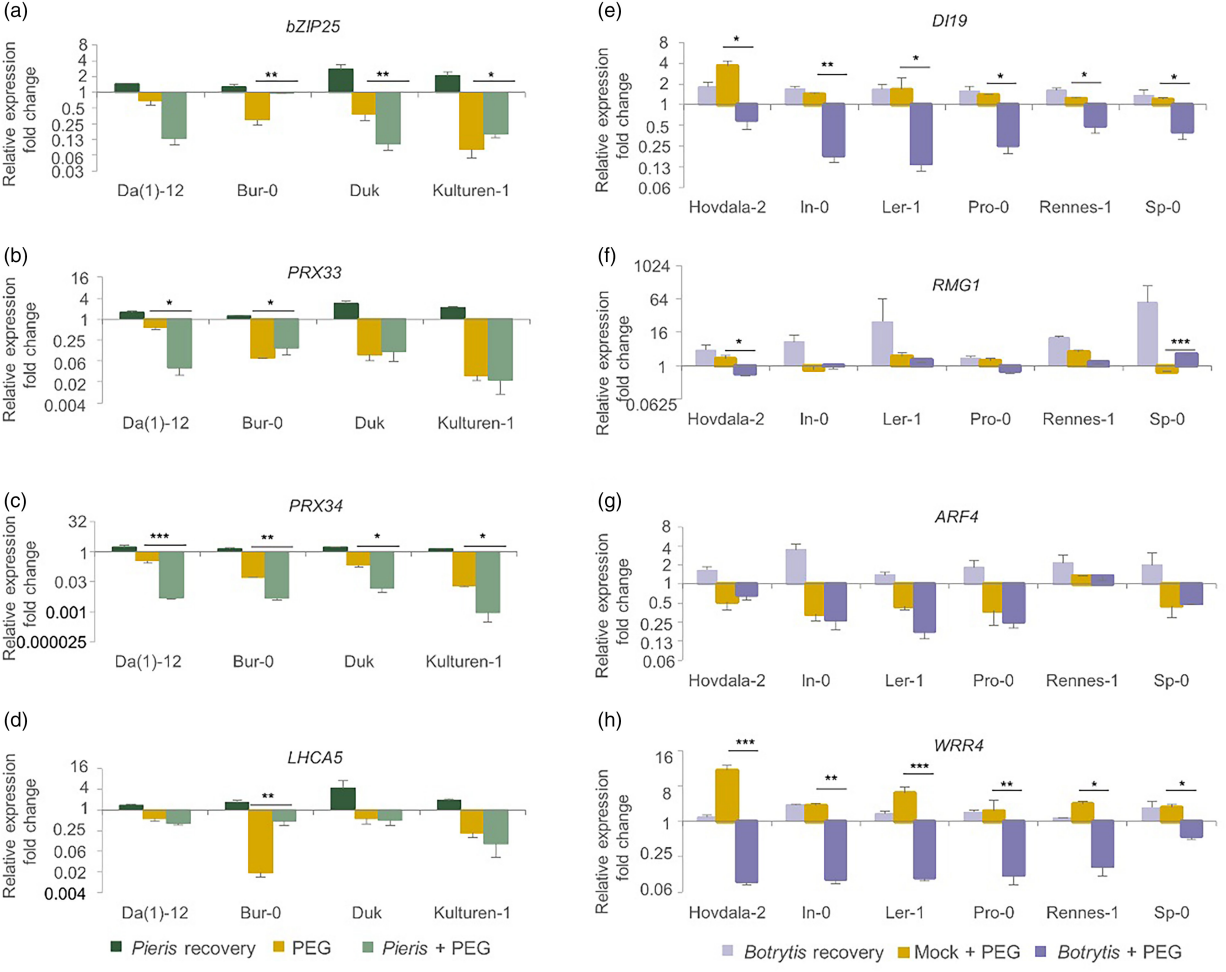
Fold changes of relative expression of (a) *bZIP25*, (b) *PRX33*, (c) *PRX34*, (d) *LHCA5*, (e) *DI19*, (f) *RMG1*, (g) *ARF4* and (h) *WRR4* in the rosettes of *Arabidopsis* accessions treated with either *Pieris* (green) or *Botrytis* (light purple), polyethylene glycol 8000 (PEG) or Mock + PEG (orange), *Pieris* + PEG (light green) or *Botrytis* + PEG (dark purple), compared to the expression under control conditions. Two‐way analysis of variance was used to compare fold changes of gene expressions under either *Pieris* + PEG or *Botrytis* + PEG to single PEG or Mock + PEG treatment. Standard error was calculated based on three plants per treatment. *p < 0.05, **p < 0.01, ***p < 0.001. *p‐*values of the main and interaction effects are provided in Table S9.

Next to the phenotypic analysis of knock‐out mutants, the expression of some of the candidate genes (*bZIP25*, *PRX33* [in LD with *PRX34*], *PRX34* and *LHCA5*) was determined in the accessions Duk, Kulturen‐1, Da(1)‐12 and Bur‐0, which showed either a susceptible or a tolerant phenotype on PLA among the 350 *Arabidopsis* accessions in the previously described GWA experiment, grown under control, *Pieris*, PEG, and *Pieris* + PEG treatments. Three‐way ANOVA was performed to test the interaction between accessions and treatments (Table S8). This showed that for *bZIP25, PRX33* and *RX34* expression there was a significant interaction between accession, *Pieris* treatment and PEG treatment (Table S9). The three genes were downregulated in all accessions grown under the *Pieris* + PEG and PEG alone treatments but upregulated upon the single *Pieris* treatment. Comparing the *Pieris* + PEG to PEG alone, *bZIP25* was downregulated in Duk and Kulturen‐1 but not found in Da(1)‐12 and Bur‐0 (Figure 6a); *PRX33* showed no significant differences in gene expression in Duk and Kulturen‐1 but was differently expressed in Da(1)‐12 and Bur‐0 (Figure 6b); *PRX34* was more strongly downregulated in all accessions under the combined stresses when compared to PEG alone (Figure 6c).

The expression levels of the *DI19*, *RMG1*, *ARF5* and *WRR4* genes in response to the *Botrytis*, PEG and *Botrytis* + PEG treatments were studied in accessions Pro‐0, Rennes‐1, Sp‐0, Hovdala‐2, In‐0 and Ler‐1, which showed extreme phenotypes in WC among the 350 *Arabidopsis* accessions in the previously described GWA experiment. Three‐way ANOVA revealed a significant interaction between accession, *Botrytis* and PEG treatments for expression fold changes of *RMG1* and *WRR4* (Table S9).

**FIGURE 7:**
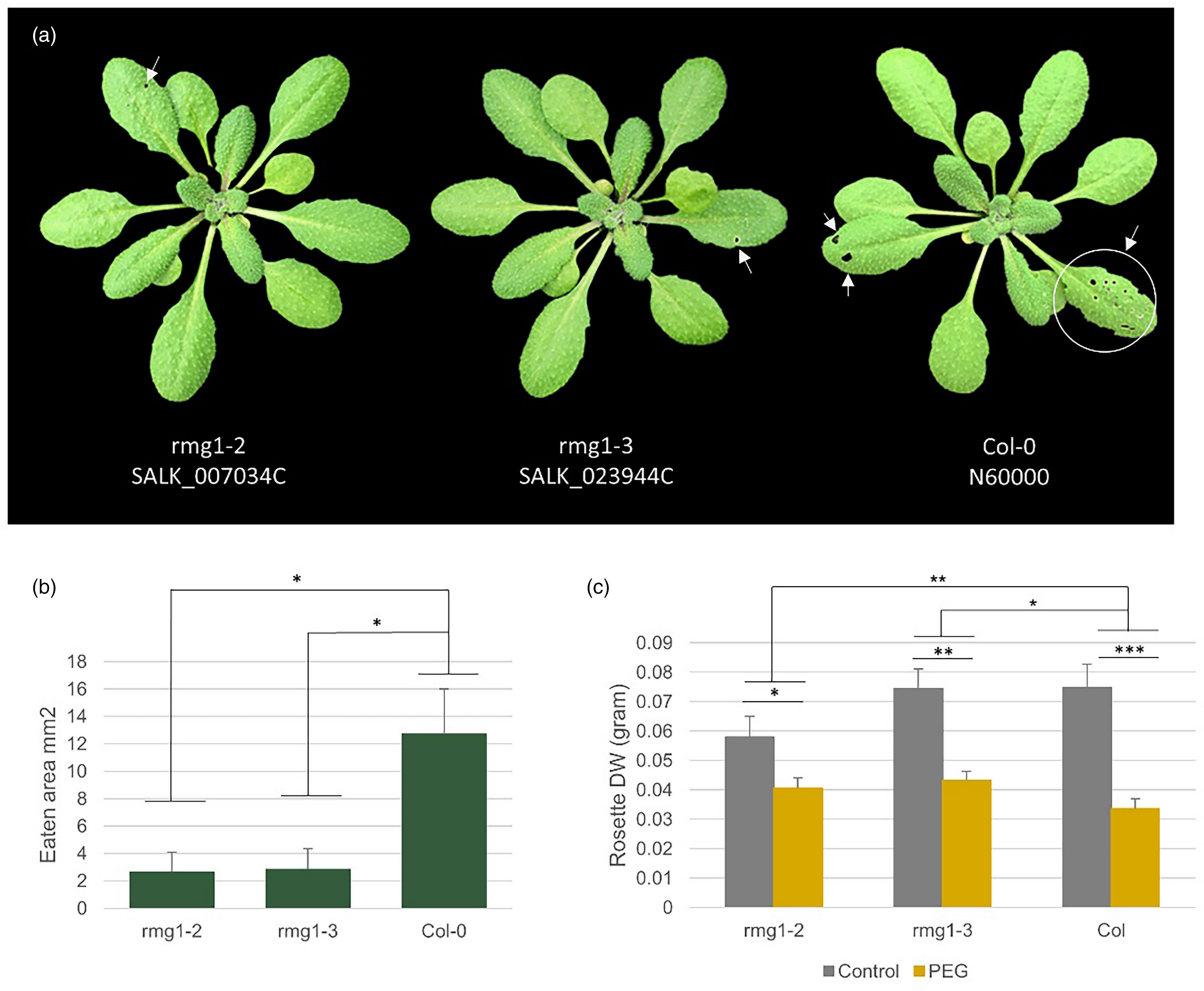
*rmg1* mutant plants showed reduced leaf eaten area by 24 h *Pieris* treatment compared with the wild type (a, b); assessed by one‐way analysis of variance [ANOVA]), and less reduced dry weight (DW) compared with the wild type (Col‐0) under polyethylene glycol 8000 (PEG) treatment (c). Standard error was calculated based on six plants per genotype per treatment. Two‐way ANOVA was used to compare the mutant to the wild type under stress treatment. *p < 0.05, **p < 0.01, ***p < 0.001. *p‐*values of the main and interaction effects are provided in Table S10.

Strong expression of *RMG1* was observed in response to *Botrytis* infection in accession Sp‐0 (Figure 6f). We did not observe a significant difference between the *rmg1* mutant and the wild type in the *Botrytis* or PEG treatments and the stress combination but noticed limited feeding intensity on the mutants by *Pieris* larvae and less reduced rosette DW of the mutants under PEG treatment (Figure 7). Upon single PEG treatment, *DI19* and *WRR4* were upregulated in all accessions, most prominently in Hovdala‐2 (Figure 6e,h). Upon *Botrytis* + PEG treatment, all accessions exhibited downregulation of *ARF4*, except Rennes‐1 (Figure 6g). Most variation for fold change expression differences was observed for *RMG1*, both with respect to differences between accessions and differences between treatments.

## 3 DISCUSSION

In our analysis, we used PEG treatment as a proxy for drought. The main reason to turn to a PEG treatment was related to the difficulties we experienced in obtaining homogeneous drought on peat‐based substrates for a large set of plants, which is essential for a reliable GWA analysis of the *Arabidopsis* HapMap population. We are aware of much more suitable systems, especially designed to screen for plant drought on soil, such as the Phenopsis system (Granier et al., 2006), but logistic issues made us instead decide to use the PEG‐mediated osmotic stress. There is also an important advantage of the PEG approach, as we could use this in our rockwool‐based growth facilities equipped with imaging systems to record plant size and thus accurately measure plant growth (Bac‐Molenaar et al., 2016; Flood et al., 2016). Using an ebb–flood system to water the rockwool blocks also allowed us to reproducibly test different PEG–nutrient solution mixtures for the most suitable osmotic potential for screening.

PEG and conventional drought by soil drying are known to cause some differences in plant responses. For example, differences in proline accumulation and leaf thickness were observed in apple; however, relative water content (RWC) and chlorophyll content were similar (Kautz et al., 2015). Therefore, and to verify that the PEG treatment induced similar, but more controlled, symptoms as observed upon conventional drought stress of plants raised on peat‐based mixtures by withholding water, we examined gene expression of five tested genes involved in the ABA‐dependent/independent regulatory system for drought responses (Shinozaki & Yamaguchi‐Shinozaki, 2007). Because we observed comparable expression profiles of these drought‐responsive genes in response to PEG and soil‐drying treatments (Figure 2), we concluded that the PEG 8000‐induced osmotic stress is sufficiently suitable to study the *Arabidopsis* response to drought. In further follow‐up research the actual response to soil drying will need to be established for the candidate genes we identified, as there are likely to be genes for which the consequence of the allelic variation affects only PEG‐induced osmotic stress response but not the conventional, soil drying, drought response.

Rather than mapping each trait for the respective treatment, we decided to map the residual values for each trait obtained after regression of the trait value of stress‐treated plants on the trait value of control‐grown plants, or after regression of combinatorial stress‐treated plants on single stress‐treated plants. The residuals represent the variations of the accessions (genotype) in response to stresses (environment) (Filiault & Maloof, 2012). Of the two GWA approaches we employed, the univariate approach yielded more associated SNPs than the ME approach. This could be due to a higher number of false positives in the univariate approach, which is known to occur when compared to the ME approach, as the latter also considers the variation within and between trait(s) (Korte et al., 2012). In addition, the ME approach has the advantage over the univariate method in that it can test multiple environment responses and thus is better at identifying SNPs representing QTL‐by‐environment interaction (Q × E). The growth traits we measured (FW, DW, WC and PLA) are likely to be genetically affected by allelic variation at many loci. For these traits, the heritability was high (Table S5). Nevertheless, the multitude of small‐effect QTLs could make it very difficult to identify the genes underlying such QTLs (Kooke et al., 2016). This was also the reason we used an arbitrary threshold of −log(*p*) = 4 to assign associated SNPs (El‐Soda et al., 2015, 2019; van Rooijen et al., 2015) instead of the very conservative Bonferroni threshold (−log(*p*) = 6.5), which corrects for multiple testing (Atwell et al., 2010). This correction assumes independence of markers, which is not the case. An alternative would be to calculate a corrected Bonferroni threshold to detect the small‐effect QTLs, focusing on the association over time instead of the strength of the association (Prinzenberg et al., 2020). If the most conservative threshold was used, we could only consider one significant SNP, m73995, close to At2g36550, mapped for rosette PLA upon *Pieris* + PEG treatment using the univariate GWA approach and the ME GWA approach. There are several SNPs in LD with the main significant SNP, all residing in the At2g36560 gene, encoding a member of the AT hook domain DNA‐binding protein family. Thus, although this is a strong candidate for the identified QTL, the Col‐0 knockout (KO) mutant for this gene did not show any difference in response to the stress treatments when compared to Col‐0. While this may have several reasons (e.g., gene redundancy or a natural mutation in Col‐0), which we did not further investigate, this did not provide strong experimental evidence to support its candidacy as the gene underlying the allelic variation.

Both the univariate and the multivariate GWA‐mapping approaches identified several QTLs that could be associated to the responses to the combined stresses. Eight were found for the response to *Pieris* + PEG, including seven SNPs on Chr 2 comprising genes with IDs At2g36550 and At2g36560, and one SNP on Chr 3 around gene At3g45910. Four overlapping SNPs were found for the response to *Botrytis* + PEG treatment, covering four loci, around genes with IDs At1g48670, At1g56280 (*DI19*), At2g13690 and a larger region comprising At5g48120–At5g48180. An earlier study reported that the *DI19* gene is expressed in seedlings, roots, rosettes, stems, flowers and siliques (Milla et al., 2006). The expression of this gene can be induced by both drought and salt stress and one of the hydrophilic proteins, AtDi19‐3, can be transcriptionally induced by the plant hormone ABA (Qin et al., 2014). In this study we observed the upregulation of the *DI19* gene in *Arabidopsis* rosettes under PEG treatment, which confirmed our conclusion that PEG treatment permits the study of drought response in *Arabidopsis*. In response to drought, *DI19* activates the expression of *PR1*, *PR2* and *PR5*, which appear to have a wider role in stress response beyond the response to pathogens (Liu et al., 2013). The *di19* mutant is reported to be more sensitive to drought and a constitutive overexpression line was more drought tolerant than wild‐type plants (Liu et al., 2013). We did not observe this for the *di19* mutant; instead, we found that the mutant was more tolerant to the PEG treatment than the wild type. The PEG treatment is less severe than imposing drought by withholding water, which could be the reason for this difference. DI19 interacts with the polycomb‐group repressor MEDEA (MEA), which mediates defence response in *Arabidopsis* and binds to the promoter of *RESISTANCE TO P. SYRINGAE 2* (*RPS2*) to suppress gene expression (Roy et al., 2018). The *di19* mutant was shown to increase upregulation of *RPS2* and was found to be resistant to *Pseudomonas syringae* pv. *tomato AvrRpt2*, while the overexpression mutant was susceptible to it. In our study, we observed the upregulation of the *DI19* gene upon *Botrytis* recovery and observed no significant differences of the mutant PLA upon *Botrytis* infection. We observed significant transcriptional downregulation of the *DI19* gene under the *Botrytis* + PEG treatment (Figure 6e). This is similar to an earlier study in which *DI19* was found to be downregulated under the combination of *Botrytis* and subsequent drought (Coolen et al., 2016). Thus, both PEG and drought, in combination with *Botrytis* treatment, have a similar antagonistic effect on the expression of *DI19*.

In LD with SNP m33786 marker, we found three SNPs mapped in three unnamed genes, At1g56290, At1g56300 and At1g56310. At1g56290 encodes a CwfJ‐like Zn‐finger DNA‐binding protein that is expressed in response to cabbage leaf curl virus infection (Ascencio‐Ibanez et al., 2008). At1g56300 encodes a chaperone DnaJ‐domain superfamily protein expressed in response to oxidative stresses. At1g56310 encodes a ribonuclease H‐like superfamily protein. None of the three SNPs mapped in these genes could explain the allelic variation. However, we did not investigate the expression of the three genes in stress conditions, nor their biological functions in response to stress treatment.

Some candidate genes in LD with associated SNPs have been reported to have a role in the *Arabidopsis* response to various stresses, but not yet in the response to *Pieris*, *Botrytis* and PEG. We therefore identified homozygous T‐DNA insertion mutants for many of these genes and determined their phenotypes in response to the studied treatments. One of those mutants was a *prx34* KO, which was found to be more susceptible to the *Pieris* stress (Figure 5a). The univariate GWA‐mapping on PLA showed another *PRX* family member, *PRX33* (*AT3G49110*), to be in LD with *PRX34*. Both genes encode cell wall peroxidases (Valerio et al., 2004). *PRX34* is more abundantly expressed than *PRX33* (Sultana et al., 2015), and the expression of *PRX33* depends on expression of *PRX34* (Daudi et al., 2012), as the *prx34* KO mutant was found to exhibit reduced expression of both *PRX33* and *PRX34* (Daudi et al., 2012; O'Brien et al., 2012). The double KO mutant *prx33prx34* (*asFBP1.1*) expresses a number of JA‐responsive genes such as *PDF1.2*, *VSP1*, *VSP2*, which are not expressed in the wild type (Mammarella et al., 2014), which makes it likely that the enhanced tolerance of the *prx34* mutant to *Pieris* is due to the herbivore‐triggered increased expression of JA‐induced defence genes. The *prx33prx34* double KO mutant also shows reduced expression of the drought tolerance gene *ETHYLENE RESPONSE FACTOR 1* (*ERF1*) (Cheng et al., 2013; Mammarella et al., 2014), which is negatively regulated by *Pieris* (De Vos et al., 2006). This would explain why the *prx34* mutant was more sensitive to the consecutive *Pieris* and PEG stress combination instead of being more tolerant as expected from the response to *Pieris* alone.

Abiotic stresses are known to also affect plant responses to biotic stresses and vice versa (Appel et al., 2014; Atkinson & Urwin, 2012; Coolen et al., 2016; Davila Olivas et al., 2016; Rejeb et al., 2014). The interaction between biotic and abiotic stress responses is often mediated by different plant hormones and the outcome can be synergistic, antagonistic or neutral (Achuo et al., 2006; Audenaert et al., 2002; Prasch & Sonnewald, 2015; Ramegowda & Senthil‐Kumar, 2015; Suzuki et al., 2014). Based on the gene expression data, two types of interaction between biotic and PEG stress were observed in our study. An antagonistic effect, which is the effect of opposing impact of the prior biotic treatment and sequential PEG treatment, was observed on the expression of *DI19* and *WRR4* genes. The two genes were upregulated under the individual *Botrytis* and PEG treatments but were downregulated under the *Botrytis* + PEG treatment (Figure 6e,h), showing the opposing interaction between the prior *Botrytis* treatment and the sequential PEG treatment. An antagonistic effect was also observed for the *arf4* mutant, which was susceptible to both *Botrytis* and PEG but not to the combined treatment (Figure 5). A synergistic effect is considered when the prior biotic treatment significantly enhances the effect of subsequential PEG on plants. This phenomenon was observed for the expression of the *PRX34* gene, which exhibited increased downregulation under the *Pieris* + PEG treatment compared with the PEG treatment alone (Figure 6c). We did not investigate the relationship between the expression profile and the phenotypic variances in *Arabidopsis* accessions in response to stresses, though such analysis might be possible through the locally linear embedding graph generator (LEGG) algorithm (Van Poecke et al., 2007).

*RMG1* was previously identified as a candidate gene underlying *Arabidopsis* natural variation for the response to several biotic and abiotic stresses including caterpillar herbivory, thrips and nematode infection, and osmotic and salt treatments, upon GWA‐mapping and *rmg1* mutant analysis (Thoen et al., 2017). *RMG1* is a primary target of RNA‐directed DNA methylation (RdDM), and its expression is controlled by pathogen‐induced Repressor of Silencing 1 (ROS1), which can suppress the RdDM activity and therefore ensure proper expression of the *RMG1* in response to infection by the pathogen *P. syringae* pv. *tomato* DC3000 (Halter et al., 2021; Yu et al., 2013). A number of WRKY family transcription factors DNA‐binding sites have also been found at the demethylated promoter region of *RMG1*, including JA‐regulated *WRKY22* (Yu et al., 2013). In this study, the *RMG1* gene was identified as a candidate based on its LD with an associated SNP in the GWAS. Although the overexpression mutant of *RMG1* was not investigated in stress conditions, the *rmg1* KO mutants revealed the broad biological function of *RMG1* in multiple stress responses (Thoen et al., 2017), as we found for herbivory and PEG treatment (Figure 7).

In this study, we identified a number of SNPs associated with combinatorial stress responses and observed both antagonistic and synergistic effect of the biotic pretreatments on sequential PEG treatment in *Arabidopsis* plants. We identified new roles of some previously reported stress‐responsive genes upon exposure to *Pieris*, *Botrytis*, PEG and the stress combinations. We did not investigate the allelic effects on significant SNPs‐associated genes in stress conditions; thus, genetic complementation tests to examine the biological function of the different alleles in response to different stress conditions need to be applied in future studies.

To conclude, this study used two GWA‐mapping approaches and identified a number of candidate genes that exhibited allelic variation in the response to single and combinatorial stresses. We validated the biological function of eight genes based on a single T‐DNA insertion mutant for each gene. These validated genes were either holding the trait‐associated SNPs identified in the GWAS or in LD with these. These genes are known to have functions in response to single stresses but not to combinatorial stresses. Antagonistic and synergistic interaction between biotic stress treatments and PEG was observed for the expression of the genes *RMG1*, *DI19* and *PRX34* in *Arabidopsis* accessions. The results of *prx34*, *di19, rmg1* and *arf4* mutants under *Pieris*, *Botrytis*, PEG and the stress combinations indicated the involvement of these genes in response to the combinatorial stress. Further studies to apply a LEGG approach may be needed to establish the relation between gene expression profile and the plant phenotypic variation in response to stresses and genetic complementation tests to examine the biological function of the different alleles of significant SNP‐associated genes in response to different stress conditions.

## 4 EXPERIMENTAL PROCEDURES

### 4.1 Plant material and growth conditions

For the GWAS, the *Arabidopsis* HapMap diversity panel, comprising a population of 341 accessions (Li et al., 2010) was used. Seeds of T‐DNA insertion mutants (Table S1) were ordered from the Nottingham *Arabidopsis* Stock Centre (http://Arabidopsis.info/), except for seeds of the *drought‐induced19* (*di19*) mutant (Liu et al., 2013), which were obtained from the authors.

Prior to the experiments, seeds were stratified at 4°C in the dark for 5 days. Thereafter, one seed per accession was sown on one Grodan Rockwool cube of 4 × 4 × 4 cm. Plants were watered three times per week (on Monday, Wednesday, and Friday) with a nutrient solution developed for *Arabidopsis* with pH 7 and EC 1.4 mS/cm. The nutrient solution consisted of 1.7 mM NH_4_^+^, 4.5 mM K^+^, 0.4 mM Na^+^, 2.3 mM Ca^2+^, 1.5 mM Mg^2+^, 4.4 mM NO_3_^−^, 0.2 mM Cl^−^, 3.5 mM SO_4_^2−^, 0.6 mM HCO_3_^−^, 1.12 mM PO_4_^3−^, 0.23 mM SiO_3_^2−^, 21 μM Fe^2+^ chelated using 3% diethylene triaminopentaacetic acid, 3.4 μM Mn^2+^, 4.7 μM Zn^2+^, 14 μM BO_3_^3−^, 6.9 μM Cu^2+^, and <0.1 μM MoO_4_^4−^. Plants were grown in a climate‐controlled growth chamber set to short‐day conditions of 10 h light/14 h dark, 21°C day/19°C night temperature, and 70% relative humidity. Irradiation was set at 200 μmol m^−2^ s^−1^.

### 4.2 Comparing drought and PEG 8000‐induced osmotic stress

Four‐week‐old Col‐0 plants were subjected to a PEG treatment by watering with nutrient solution that contained 7.7% wt/vol PEG 8000 for 6 days, and compared to soil‐grown Col‐0 plants subjected to drought by withholding water for 6 days. The expression of five drought‐responsive genes; *RD26* (*At4g27410*), *MYC2* (*At1g32640*), *RD29b* (*At5g52300*), *RD29a* (*At5g52310*), and *P5CS1* (*At2g39800*) (Shinozaki & Yamaguchi‐Shinozaki, 2007) was determined by reverse transcriptase‐quantitative real‐time PCR (RT‐qPCR) under both treatments. Primers of all investigated genes and the five drought‐responsive genes are provided in Table S2.

RNA was extracted from *A. thaliana* rosettes (Oñate‐Sánchez & Vicente‐Carbajosa, 2008). cDNA was synthesized from 800 ng of total RNA using an iScript cDNA synthesis kit (Bio‐Rad). cDNA was diluted 10 times before qPCR. The gene with ID At3g15930 was selected as reference for RT‐qPCR as the most stably expressed across different drought, osmotic, and *Botrytis* treatments based on the transcriptome database Genevestigator (refgenes.org/rg). The qPCR was performed using iQ SYBR Green Supermix (cat. no. 170‐8885) on a Bio‐Rad CFX96 real‐time PCR system set at 95°C for 4 min, followed by 40 cycles of 95°C for 10 s and 55°C for 30 s. Relative gene expression was calculated using the 2^(−ΔΔ*C*t)^ method (Livak & Schmittgen, 2001). The standard errors were calculated from at least three plants per accession.

### 4.3 Preparation of *P. rapae* and *B. cinerea* for treatments

*P. rapae* caterpillars were reared on cabbage plants (*Brassica oleracea* var. *gemmifera* ‘Cyrus’) in a climate‐controlled room (set to 20–24°C, 50%–70% relative humidity, 16 h light/8 h dark photoperiod). Butterflies were supplied with a solution of 20% honey and 10% sucrose as food. Inbreeding of the population was minimized by adding wild butterflies and caterpillars to the existing population. After starving for 1 h, first‐instar (L1) larvae were placed on *Arabidopsis* leaves using a fine paint brush (Coolen et al., 2016).

*B. cinerea* B05.10 (Staats & Van Kan, 2012) was grown on half‐strength potato dextrose agar (PDA; Difco) plates containing penicillin (100 μg/mL) and streptomycin (200 μg/mL) for 2 weeks at room temperature. Spores were collected, filtered through glass wool, and resuspended in half‐strength potato dextrose broth (PDB; Difco) to a final density of 10^5^ spores/mL. After 3 h of incubation period, the spores were used for inoculation by applying two 5‐μL droplets on one *Arabidopsis* leaf (Coolen et al., 2016).

### 4.4 Single and combinatorial stresses imposed on the HapMap population

For the single osmotic treatment, 19‐day‐old plants were irrigated with nutrient solution that contained 7.7% wt/vol PEG 8000 for 6 days. For combinatorial stress, 17‐day‐old plants were exposed to one *Pieris* first‐instar larva, deposited on one of the rosette leaves, or inoculated with two 5‐μL droplets of a *Botrytis* spore suspension, and incubated for 1 day at high humidity. The next day, the caterpillars were removed, or plants were moved to control humidity conditions, and the plant continued to grow in the control conditions for 1 day, followed by irrigation with nutrient solution that contained 7.7% wt/vol PEG 8000 for 6 days (“*Pieris* + PEG” and “*Botrytis +* PEG”). Plants that did not receive any stress treatment were used as a control (Figure 2a). Plants that were subjected to the single PEG treatment without any pretreatment are called “Mock + PEG”.

To test T‐DNA mutants, for the single *Pieris* treatment, 17‐day‐old plants were exposed to either one *Pieris* first‐instar larva or two 5‐μL droplets of *Botrytis* spore suspension for 1 day. Thereafter, the caterpillars were removed and plants were allowed to grow in control conditions for 6 days (“*Pieris*” and “*Botrytis*”) (Figure 2b).

Extreme accessions from the HapMap population were selected based on their DW and tested for gene expression using the same design as described above.

### 4.5 Plant phenotyping

The experimental setup to screen the HapMap diversity panel used for phenotyping was according to a randomized block design, with two blocks per treatment, each containing one full set of genotypes, and with two treatments in the same growth room. For all plants used for GWA mapping, projected rosette leaves area (PLA) was measured. For the sequential *Pieris* and PEG treatment, plants were measured directly before *Pieris* pretreatment (T1), after the *Pieris* pretreatment (T2), and 6 days after the PEG treatment (T3) (Figure 2). For the sequential *Botrytis* and PEG treatment, the rosettes of too many plants were overlapping and the data was not used for mapping. Instead, rosette fresh weight (FW) and dry weight (DW) were measured from the single PEG and the sequential *Botrytis* and PEG treatments (Figure 2). Rosette water content (WC) was calculated using the formula

$$\mathrm{WC}=\frac{\text{fresh weight}\;-\;\mathrm{dry}\;\text{weight}}{\text{fresh weight}}.$$

### 4.6 Genome‐wide association mapping

For PLA, residuals were calculated from the regression analysis between the PLA measured after *Pieris* treatment (T2) and the PLA measured after PEG treatment (T3), whereas residuals of DW and WC were calculated from the regression between the two treatments, that is, *Botrytis* + PEG and Mock + PEG. These residual values were used for mapping. For ME‐GWA‐mapping (Korte et al., 2012) a minor allele frequency (MAF) of 0.05 was used and an arbitrary threshold of −log(*p*) = 4 was used as described earlier by El‐Soda et al. (2019). Univariate GWA‐mapping was performed using the scan_GLS software as described earlier (Kang et al., 2010; Kruijer et al., 2014). In brief, this approach involves performing generalized least squares (GLS) calculations conditional on the variance components, which were estimated in the model without markers and can efficiently handle genetically identical individuals. Thereafter, SNPs with MAF <0.05 were excluded. The proportion of explained phenotypic variance was the criterion to identify significant SNPs as described by Sun et al. (2010). The information of SNPs in the LD with the significant SNPs were identified by the online LD tool (http://dev3.ab.wur.nl/AthaLD), as well as the information of coding sequence substitution and amino acid substitution.

### 4.7 Selection of candidate genes

For each of the identified QTLs a so‐called haplotype analysis was performed, based on each SNP in the region found to be associated with the trait, or a series of SNPs in LD, if possible. This distinguishes two alleles, the Col‐0 reference allele and the non‐reference allele, per SNP. Phenotypes are expected to differ per group of genotypes sharing either the Col‐0 or the non‐reference gene. This analysis was used to confirm the found SNP‐phenotype association. Gene ontology annotation of selected candidate genes for the QTLs identified after the GWA analysis was found in The *Arabidopsis* Information Resource (TAIR; http://www.arabidopsis.org/tools/bulk/go/index.jsp). Genes characterized by one or more of the following criteria were considered as candidates: (1) genes known to be responsive to a biotic or abiotic stimuli and expressed under abiotic stress and hormonal (JA and ABA) treatments; (2) genes with reported physiological functions in response to stress conditions and expressed under abiotic stresses and hormonal (JA and ABA) treatments (http://bar.utoronto.ca/efp/cgi‐bin/efpWeb.cgi); (3) genes with reported physiological functions in response to stress conditions and responsive to biotic and abiotic stimuli. T‐DNA mutants of the candidate genes were ordered from the Nottingham *Arabidopsis* Stock Centre if available as homozygotes.

### 4.8 Statistical analysis

The broad‐sense heritability (*H* ^2^) was calculated for each trait as the ratio between the genetic variance (*δ* ^2^ *g*) and the total phenotypic variance (*δ* ^2^ *ph*) where *δ* ^2^ *ph* = *δ* ^2^ *g* + *δ* ^2^ *e* and *δ* ^2^ *e* is the environmental variation, that is, the variance between replications of each line

$$H^{2}=\frac{\delta^{2}g\;}{\delta^{2}g+\delta^{2}e}.$$

Pearson's correlation coefficient *r*, one‐way and two‐way ANOVAs were performed using GenStat for Windows 16th Edition (VSN International Ltd). Student's *t* test was used to compare the growth of the same accession between treatments. Three‐way ANOVA was performed as described by Sokal (1981). Statistical tests of FW, DW and WC were performed on five replicates per accession, whereas gene expression was performed on three replicates per accession.

## Supporting information


**Figure S1.** Results of significant SNPs (−log_10_(*p*) > 4) from univariate and multi environment (ME) GWA‐mapping showed in Manhattan plots. Blue and red colour indicates each chromosome. Arrow indicates the single‐nucleotide polymorphism (SNP)(s) residing genes. Dashed horizontal line indicates threshold at −log_10_(*p*) = 4. (a–c) Manhattan plots of univariate GWA‐mapping of (a) projected rosette area (PLA) residuals (regression analysis between *Pieris* + PEG vs. *Pieris*), (b) rosette dry weight (DW) residuals and (c) water content (WC) residuals (regression analysis between of *Botrytis* + PEG vs. Mock + PEG). (d–f) Manhattan plot ME GWA‐mapping of (d) PLA for *Pieris* + PEG responses, (e) DW and (f) WC for *Botrytis* + PEG responses.

**Figure S2.** Effect of two alleles (Col‐0 like and non‐Col‐0 like) of 11 significant single‐nucleotide polymorphisms (SNPs) in response to *Pieris* and polyethylene glycol 8000 (PEG), compared to single PEG treatment. (a) m73984 associated to unnamed gene AT2g36540, (b) m73991 associated to unnamed gene At2g36550, (c–h) m73992–m73997 associated to unnamed gene At2g36560, (i) m74101 associated to gene At2g36580, (j, k) m74017 and m74024 are associated to gene At2g36590. In all figures, *y*‐axis represents project rosette area (PLA) ratio under the *Pieris* + PEG treatment versus the single *Pieris* treatment. Student’s *t* test was sued to test the significance of the effects of the two types alleles on the combinatorial stress responses. *p < 0.05, **p < 0.01, ***p < 0.001.

**Figure S3.** Effects of two alleles (Col‐0 like and non‐Col like) of single‐nucleotide polymorphisms (SNPs). (a) The combination of four SNPs (m73984–m74024), (b) the combination of six SNPs (m200137–m200389), (c) the combination of SNPs m200317 and m200318, (d) SNP 200389, (e) SNP m60883, on rosette water content (WC) under the *Botrytis* + polyethylene glycol 8000 (PEG) treatment, compared to the single PEG treatment. Student’s *t* test was sued to test the significance of the effects of the two types alleles on the combinatorial stress responses. No significant difference between the Col‐0 like allele and the non‐Col allele was observed.

**Figure S4.** Projected rosette area (PLA) of *Arabidopsis* Col‐0 and *di19* mutant in control, *Botrytis*, polyethylene glycol 8000 (PEG), and the *Botrytis* and PEG conditions. Student’s *t* test was used to compare the PLA between Col‐0 and *di19* mutant. *p < 0.05, **p < 0.01, ***p < 0.001.

**Table S1.** T‐DNA mutants and their corresponding candidate genes. * indicates the same mutant was used in other studies, and the transcripts level of the knock‐out gene has determined in the study.

**Table S2.** Primers of five drought‐responsive genes (*RD29a, RD29b, P5CS1, RD26, MYC2*) and selected candidate genes for reverse transcription‐quantitativePCR.

**Table S3.** Pearson’s correlation coefficients when comparing rosette fresh weight (FW) for each indicated treatment.

**Table S4.** Broad‐sense heritability of different traits of the *Arabidopsis* HapMap set of accessions grown under control and stress treatments.

**Table S5.** Two‐way analysis of variance results shows significant main effect of Accessions and Treatments on water content (WC) and projected rosette area (PLA). In all tests, no significant interactions between Accessions and Treatments were observed.

**Table S6.** Univariate genome‐wide association mapping of projected rosette area (PLA), dry weight (DW), water content (WC) identified significant single‐nucleotide polymorphisms (SNPs) (−log_10_(*p*) > 4) in response to the either *Pieris* + polyethylene glycol 8000)(PEG) or *Botrytis* + PEG responses. Gene1 and gene2 are two genes associated to the same significant SNP. MAF = minor allele frequency.

**Table S7.** Multi‐environment (ME) genome‐wide association‐mapping identified significant single‐nucleotide polymorphisms (SNPs) (−log_10_(*p*) > 4) showed Q × E interaction for *Pieris* + polyethylene glycol 8000 (PEG) and *Botrytis* + PEG stress responses.

**Table S8.** Three‐way analysis of variance for testing interaction between accessions and treatments.

**Table S9.** Results of three‐way analysis of variance to teste the interaction between accessions and treatment on genes *bZIP25, PRX3, PRX34, LHCA5, DI19*, *RMG1*, *ARF4* and *WRR4*.

**Table S10.** Results of one‐way and two‐way analysis of variance to test the interaction between mutants and treatment exposure to *Pieris* and polyethylene glycol 8000 (PEG).

## Data Availability

The data that support the findings of this study are available from the corresponding author upon reasonable request.
