# Supplementary figures and images for: Genome‐wide association analysis reveals genes controlling an antagonistic effect of biotic and osmotic stress on Arabidopsis thaliana growth
Source: Mol Plant Pathol. 2024 Mar 9;25(3):e13436. doi: 10.1111/mpp.13436 (PMC10924621; doi:10.1111/mpp.13436)

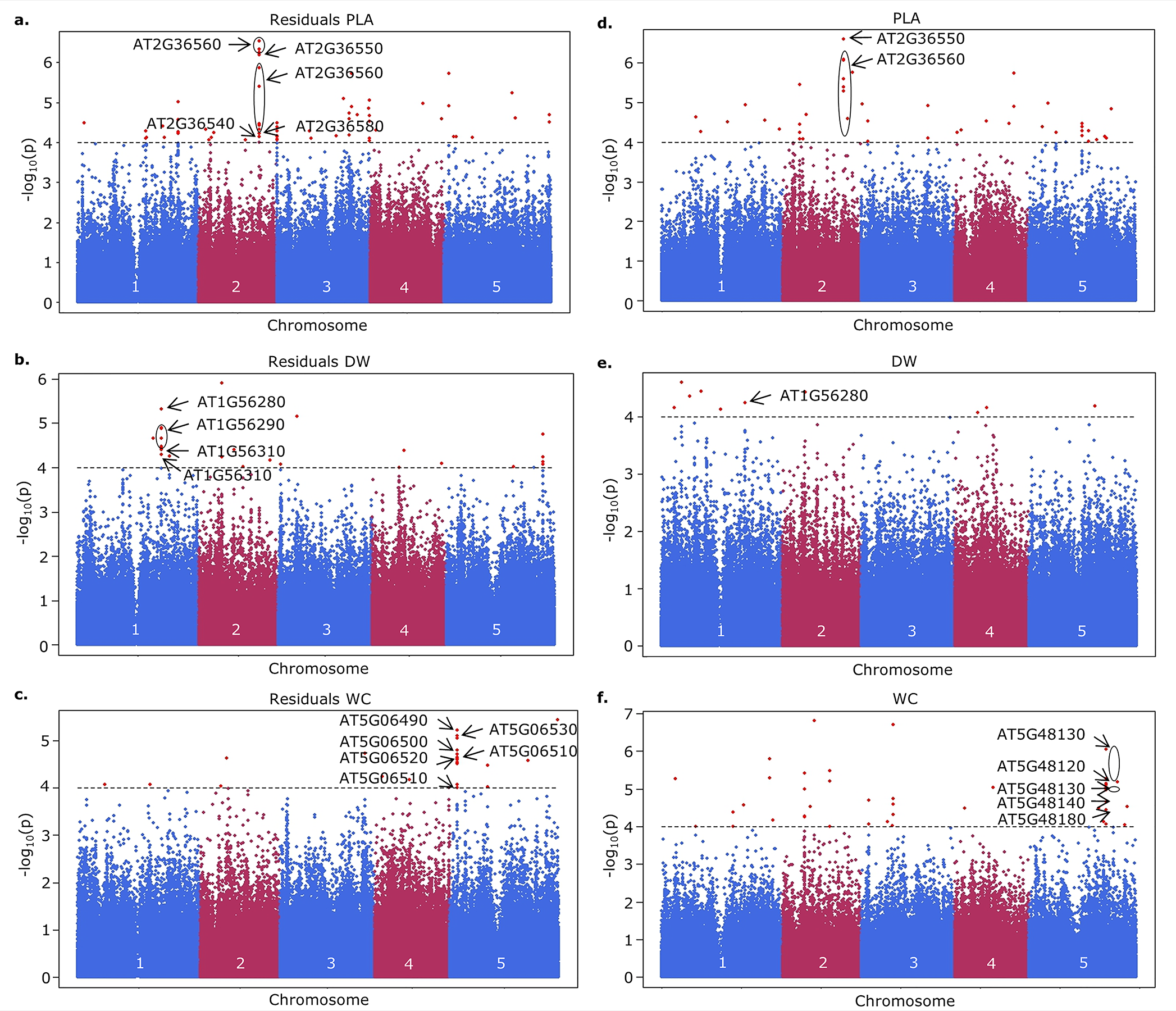

Supplement: Supplementary file 1 — Figure S1. Results of significant SNPs (−log10(p) > 4) from univariate and multi environment (ME) GWA‐mapping showed in Manhattan plots. Blue and red colour indicates each chromosome. Arrow indicates the single‐nucleotide polymorphism (SNP)(s) residing genes. Dashed horizontal line indicates threshold at −log10(p) = 4. (a–c) Manhattan plots of univariate GWA‐mapping of (a) projected rosette area (PLA) residuals (regression analysis between Pieris + PEG vs. Pieris), (b) rosette dry weight (DW) residuals and (c) water content (WC) residuals (regression analysis between of Botrytis + PEG vs. Mock + PEG). (d–f) Manhattan plot ME GWA‐mapping of (d) PLA for Pieris + PEG responses, (e) DW and (f) WC for Botrytis + PEG responses. [file MPP-25-e13436-s010.tif]

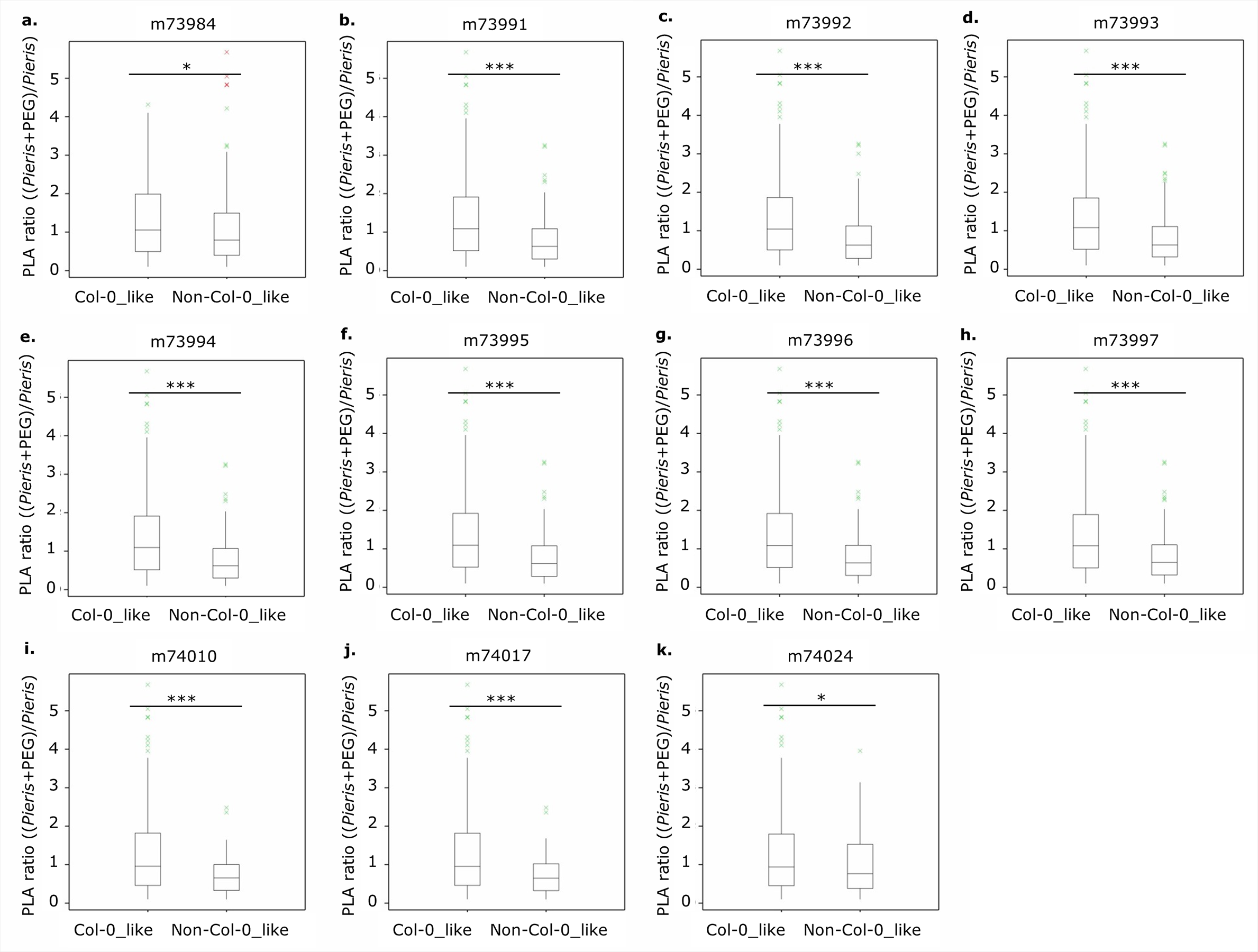

Supplement: Supplementary file 2 — Figure S2. Effect of two alleles (Col‐0 like and non‐Col‐0 like) of 11 significant single‐nucleotide polymorphisms (SNPs) in response to Pieris and polyethylene glycol 8000 (PEG), compared to single PEG treatment. (a) m73984 associated to unnamed gene AT2g36540, (b) m73991 associated to unnamed gene At2g36550, (c–h) m73992–m73997 associated to unnamed gene At2g36560, (i) m74101 associated to gene At2g36580, (j, k) m74017 and m74024 are associated to gene At2g36590. In all figures, y‐axis represents project rosette area (PLA) ratio under the Pieris + PEG treatment versus the single Pieris treatment. Student’s t test was sued to test the significance of the effects of the two types alleles on the combinatorial stress responses. *p < 0.05, **p < 0.01, ***p < 0.001. [file MPP-25-e13436-s012.tif]

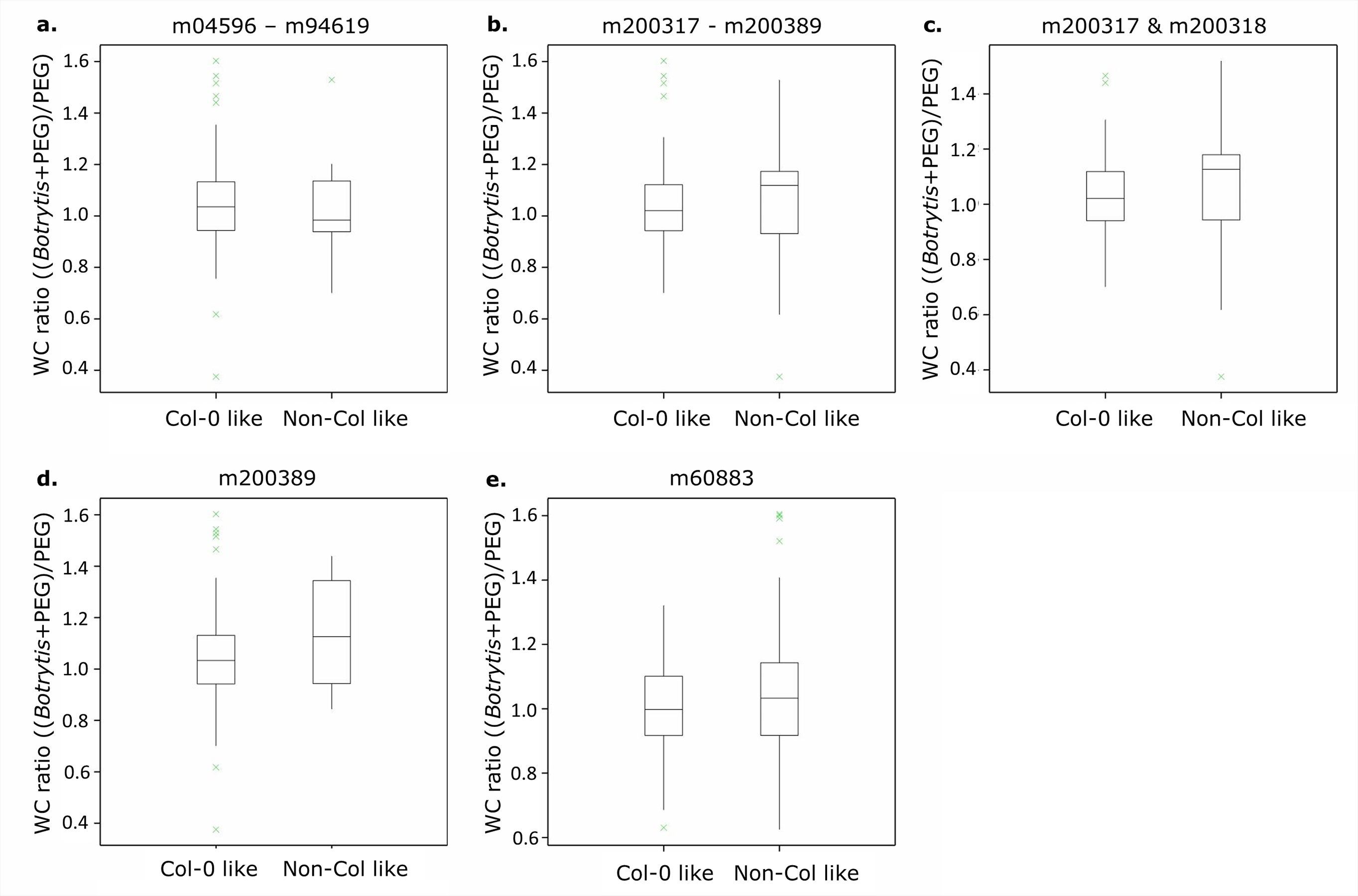

Supplement: Supplementary file 3 — Figure S3. Effects of two alleles (Col‐0 like and non‐Col like) of single‐nucleotide polymorphisms (SNPs). (a) The combination of four SNPs (m73984–m74024), (b) the combination of six SNPs (m200137–m200389), (c) the combination of SNPs m200317 and m200318, (d) SNP 200389, (e) SNP m60883, on rosette water content (WC) under the Botrytis + polyethylene glycol 8000 (PEG) treatment, compared to the single PEG treatment. Student’s t test was sued to test the significance of the effects of the two types alleles on the combinatorial stress responses. No significant difference between the Col‐0 like allele and the non‐Col allele was observed. [file MPP-25-e13436-s005.tif]

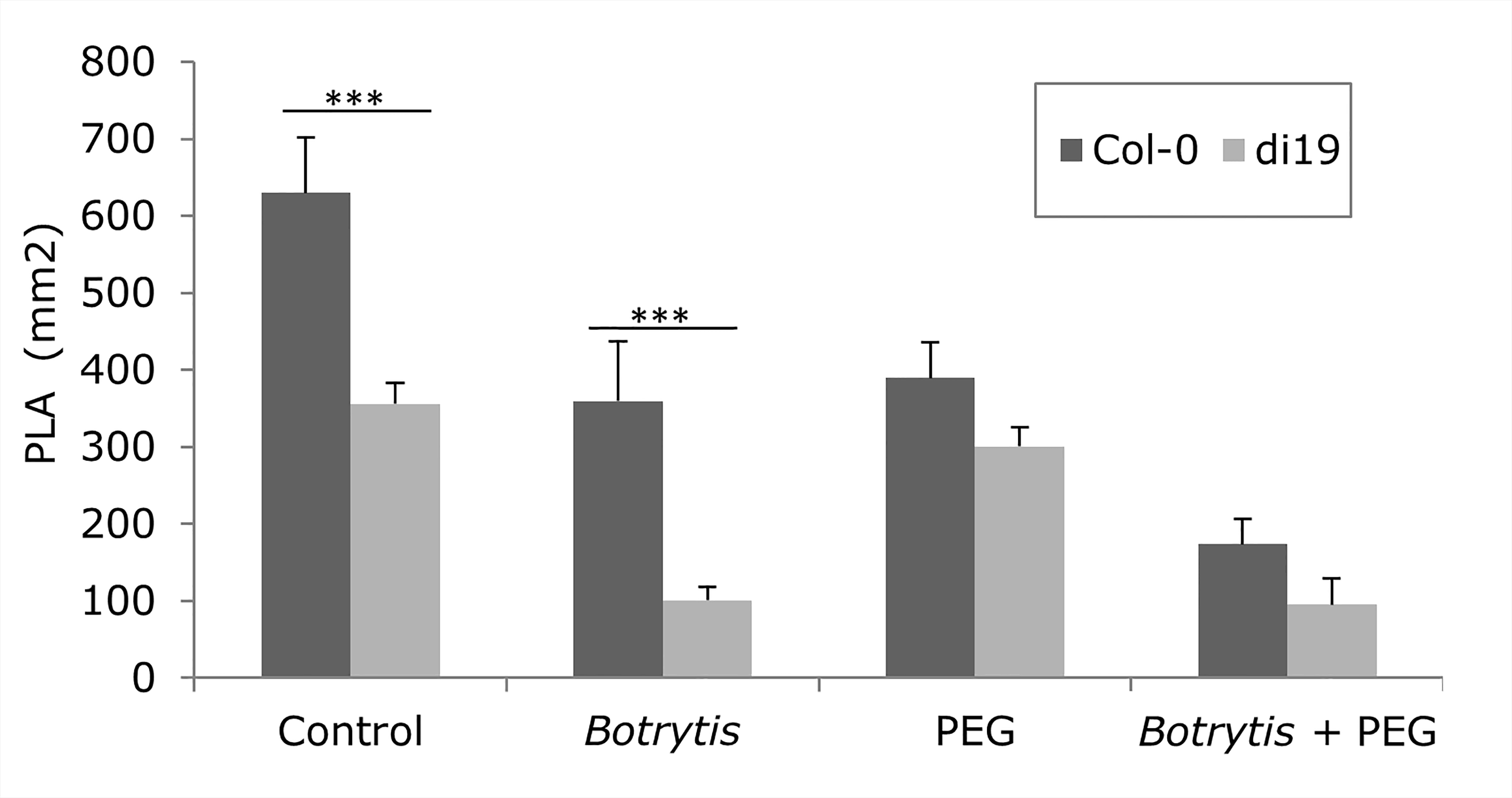

Supplement: Supplementary file 4 — Figure S4. Projected rosette area (PLA) of Arabidopsis Col‐0 and di19 mutant in control, Botrytis, polyethylene glycol 8000 (PEG), and the Botrytis and PEG conditions. Student’s t test was used to compare the PLA between Col‐0 and di19 mutant. *p < 0.05, **p < 0.01, ***p < 0.001. [file MPP-25-e13436-s003.tif]
